# Genetic Nrf2 Overactivation Inhibits the Deleterious Effects Induced by Hepatocyte-Specific c-met Deletion during the Progression of NASH

**DOI:** 10.1155/2017/3420286

**Published:** 2017-06-06

**Authors:** Pierluigi Ramadori, Hannah Drescher, Stephanie Erschfeld, Athanassios Fragoulis, Thomas W. Kensler, Christoph Jan Wruck, Francisco Javier Cubero, Christian Trautwein, Konrad L. Streetz, Daniela C. Kroy

**Affiliations:** ^1^Department of Medicine III, University Hospital RWTH Aachen, Aachen, Germany; ^2^Department of Anatomy and Cell Biology, University Hospital RWTH Aachen, Aachen, Germany; ^3^Department of Pharmacology & Chemical Biology, School of Medicine, University of Pittsburgh, Pittsburgh, PA, USA; ^4^Department of Immunology, Complutense University School of Medicine, Madrid, Spain; ^5^12 de Octubre Health Research Institute (imas12), Madrid, Spain

## Abstract

We have recently shown that hepatocyte-specific c-met deficiency accelerates the progression of nonalcoholic steatohepatitis in experimental murine models resulting in augmented production of reactive oxygen species and accelerated development of fibrosis. The aim of this study focuses on the elucidation of the underlying cellular mechanisms driven by Nrf2 overactivation in hepatocytes lacking c-met receptor characterized by a severe unbalance between pro-oxidant and antioxidant functions. Control mice (c-met^fx/fx^), single c-met knockouts (c-met^Δhepa^), and double c-met/Keap1 knockouts (met/Keap1^Δhepa^) were then fed a chow or a methionine-choline-deficient (MCD) diet, respectively, for 4 weeks to reproduce the features of nonalcoholic steatohepatitis. Upon MCD feeding, met/Keap1^Δhepa^ mice displayed increased liver mass albeit decreased triglyceride accumulation. The marked increase of oxidative stress observed in c-met^Δhepa^ was restored in the double mutants as assessed by 4-HNE immunostaining and by the expression of genes responsible for the generation of free radicals. Moreover, double knockout mice presented a reduced amount of liver-infiltrating cells and the exacerbation of fibrosis progression observed in c-met^Δhepa^ livers was significantly inhibited in met/Keap1^Δhepa^. Therefore, genetic activation of the antioxidant transcription factor Nrf2 improves liver damage and repair in hepatocyte-specific c-met-deficient mice mainly through restoring a balance in the cellular redox homeostasis.

## 1. Introduction

Formation of reactive oxygen species (ROS) has been considered classically a pathophysiological phenomenon critically involved in the progression from simple hepatic steatosis to steatohepatitis. Upon triglyceride accumulation, cellular compartments responsible for lipid catabolism such as mitochondria and lysosomes increase their activity with a consequent generation of free radicals that trigger molecular signals leading to cell death and release of proinflammatory mediators. In this context, the use of antioxidant buffering against the generation of ROS has been shown to partially reduce the progression of nonalcoholic steatohepatitis. A growing body of evidence indicates the HGF (hepatocyte growth factor)/c-met axis as a molecular pathway linked to the control of the cellular redox homeostasis. However, data concerning the consequences of HGF stimulation on the cellular generation of free radicals are still quite controversial. Whereas in primary cell lines and tissues such as cardiomyocytes [1] and neurons [2], stimulation with HGF was shown to dampen ROS production and to reduce oxidative stress-dependent apoptosis; in cancer cell lines [3] and other in vitro conditions [4], HGF treatment resulted in augmented cell motility accompanied by increased ROS production. Our group and others recently showed that disruption of c-met functionality aggravates the onset of NASH through the impairment of mechanisms regulating cell sensitivity to lipotoxicity, ROS production, and cell proliferation [5, 6]. In particular, data emerging from genomic array analysis clearly indicated an aberrant regulation of a pattern of genes responsible for increased pro-oxidant environment, amongst them the transcription factor Nrf2 (nuclear factor erythroid-derived 2-like 2) [5].

Under conditions of oxidative or electrophilic stress, Nrf2 degradation is inhibited through oxidant-dependent modifications of specific cysteine residues within Keap1, a protein that under quiescent conditions facilitates the marking of Nrf2 for degradation via the proteasome [7]. It is now well established that stabilization and activation of Nrf2 through pharmacological or genetic targeting improves cellular redox homeostasis and survival through transcriptional upregulation of antioxidant and detoxifying genes [8]. In line with these studies, we recently showed that activation of Nrf2 in hepatocytes afforded by genetic deletion of Keap1 was able to reduce triglycerides accumulation and ROS generation in mice subjected to experimental models of NASH [9]. This former observation leads to the generation of a double knockout mouse lacking simultaneously the receptor c-met and Keap1 specifically in hepatocytes to investigate the effects of Nrf2 overexpression in cells displaying an impaired control of the redox functions. In fact, the purpose of this study pointed towards uncovering a bridge between the HGF/c-met axis and the Keap1/Nrf2 system in the context of metabolic liver disturbances (schematic overview in Suppl. Fig. 4 available online at https://doi.org/10.1155/2017/3420286). The results emerging indicated that overexpression of Nrf2 was able to suppress the levels of liver steatosis and fibrosis in c-met-deficient hepatocytes to the level of the control group, with a drastic reduction of triglyceride (TG) accumulation and ROS production. Considering that preliminary clinical data indicate a reduction of c-met expression in patients diagnosed with NASH, this study provides further evidence for strategies for therapeutic interventions in this field.

## 2. Materials and Methods

### 2.1. Animals and Experimental Model

Hepatocyte-specific Keap1 knockout mice were generated by breeding Keap1-floxed mice with albumin-Cre (Alb-Cre) C57BL/6 transgenic mice as previously described [10]. Similarly, floxed wild type (c-met^fx/fx^) and hepatocyte-specific conditional c-met knockout (c-met^Δhepa^) mice were generated under control of a postnatal activated albumin promoter (C57BL/6), as indicated elsewhere [11]. These two strains were then crossed to generate double mutant c-met/Keap1 (met/Keap1^Δhepa^) harboring the same genetic background. Male age-matched wild type (c-met^fx/fx^) and littermate hepatocyte-specific conditional c-met-knockout (c-met^Δhepa^) were then cohoused with genetically derived met/Keap1^Δhepa^ in 12-hour light/dark cycle and allowed to free food and water. At the age of 8–10 weeks, the animals were split in three groups (*N* = 5) and fed a chow and MCD diet (E15652-94, ssniff Spezialdiäten GmbH), respectively, for a period of 4 weeks. Food intake and body weights were measured weekly, and all animals consumed similar amounts of diets. At the end of the indicated time point, blood and liver samples were collected, fixed in formalin, and cryopreserved for biochemical and histological analyses. All animal experiments were carried out in accordance with the regulations of the German legal requirements on laboratory animal care (LANUV).

### 2.2. Histological and Morphological Analyses

After explant, liver tissues were fixed in formaldehyde-buffered solution for 24 h and then embedded in paraffin. Blocks containing preserved hepatic tissues were then cut into 5 *μ*m sections and stained with hematoxylin and eosin (H&E) for microscopic examination and morphological analyses. Similarly, 8 *μ*m thin liver sections were stained, upon deparaffinization, with a Sirius red/picric acid (Sigma-Aldrich) solution for 1 h as previously described [9]. Upon dehydration and mounting, photomicrographs of stained sections were randomly taken in a 200x magnification and positive areas were quantified using the open source software ImageJ.

### 2.3. Serum and Liver Biochemical Measurements

Blood samples were collected and centrifuged in heparin-embedded tubes, and serum transaminase levels (ALT/AST) were measured according to the standard procedures of the Central Laboratory Facility of the University Hospital RWTH of Aachen as index of liver damage. For the evaluation of intrahepatic triglyceride content, liver samples were homogenized in a specific Tris buffer (10 mM Tris, 2 mM EDTA, 0.25 M sucrose, and pH 7.5) and successively processed according to the manufacturer's instructions of a commercial colorimetric kit (10724600, Human Diagnostics). For biochemical quantification of hepatic collagen deposition, 50 mg of liver samples were homogenized in 1 ml of 6 N HCl solution and incubated o.n. at 110°C. Homogenates were then treated with a chloramine-T solution and incubated with Ehrlich's reagent to measure hydroxyproline content by biochemical photometric assay as indicated in previous publication [12].

### 2.4. RNA Isolation and qPCR Analysis

Total RNA from hepatic tissue was isolated with peqGold RNAPure solution (30–1020, PeqLab, VWR, Germany) according to the manufacturer instructions. An amount of 500 ng of isolated RNA was transcribed into cDNA using the Omniscript Reverse Transcription kit (205111, Qiagen). Quantitative qPCR was performed by using a Real-time PCR System 7300 (Applied Biosystems) and Fast SYBR Green Master Mix qPCR (Thermo Fisher Scientific). Relative gene expression was calculated using the 2^−ΔΔCt^ quantification formula normalizing each gene with the expression of the housekeeping gene 18S (ribosomal subunit). The primers used in this study are reported in Suppl. Table 1.

### 2.5. Western Blot Analyses

Total hepatic homogenates were performed by lysing liver samples with an Ultra-Turrax homogenizer in Tris/HCl-lysis buffer containing inhibitors of proteases and phosphatases as described before [9]. Fifty to eighty micrograms of total lysates were denatured in Laemmli sample buffer and separated in 10% and 12% SDS-PAGE gel. Upon electrophoresis and transfer blotting, Ponceau-Red staining was used to verify transfer efficiency and equal protein loading. Membranes were then blocked in blocking buffer and incubated overnight at 4°C with primary antibodies (Suppl. Table 2). Therefore, secondary antibodies were incubated on the membranes for one hour at room temperature. The following secondary antibodies have been used in this study: HRP-linked anti-rabbit immunoglobulin G (7074, Cell Signaling) and HRP-linked anti-mouse immunoglobulin G (sc-2005, Santa Cruz). Enhanced chemiluminescence (ECL) method was used to detect protein bands and the software ImageJ was employed for densitometric analysis of band intensity.

### 2.6. FACS Analysis of Myeloid and Lymphoid Hepatic Infiltrates

A nonparenchymal cell fraction from whole liver extracts was isolated upon collagenase and mechanical digestion followed by Percoll (GE Healthcare Life Sciences) gradient centrifugation as previously described [5]. In parallel, blood samples were collected in EDTA-containing tubes and treated with red blood cell lysis buffer (PharmLyse, BD Biosciences, Germany). Upon removal of red bodies and centrifugation, immune cells were incubated with fluorochrome-conjugated antibodies and characterized according to two different panels, a myeloid panel: CD45-BV510 (103138, BioLegend), 7AAD-PE-Cy5-YG (420404, BioLegend), CD11b-BV711 (101242, BioLegend), F4/80-APC (17-4801-82, eBiosciences), MHC2-Alexa700 (107622, BioLegend), CD11c-PE-Cy7 (25-0114-81, eBiosciences), and Ly6G-FITC (551460, BD Pharmingen) and a lymphoid panel: CD45-BV510 (103138, BioLegend), 7AAD-PE-Cy5-YG (420404, BioLegend), CD3-PE-Cy7 (25-0031-82, eBiosciences), CD4-FITC (11-0041-85, eBiosciences), CD8-PerCpCy5.5 (126610, BioLegend), and NK1.1-BV711 (108745, BioLegend). Labeled cells were then subjected to flow cytometry using a BD Canto II (BD Biosciences) and relative cell numbers were analyzed using FlowJo software (Tree Star).

### 2.7. Immunohistochemistry and TUNEL Assay

For 4-HNE immunostaining, paraffin-embedded sections were used. Upon antigen retrieval in sodium citrate buffer, endogenous peroxidases were inhibited through incubation with 3% H_2_O_2_ in PBS buffer for 10 minutes. Blocking was performed by incubating the sections with 5% goat serum in PBS buffer for 1 hour. Sections were then incubated with primary antibody in blocking solution overnight at 4°C and successively 1 hour at room temperature with secondary antibody (anti-mouse biotinylated). Antigen was visualized using a peroxidase substrate DAB kit (di-amino benzidine) (DAKO). For immunofluorescence staining, hepatic 5 *μ*m cryosections were fixed in 4% paraformaldehyde-buffered solution. Blocking was performed by incubating the sections with 0.2% BSA in PBS buffer for 5 minutes. Then sections were incubated with primary antibody for 1 h at RT in PBS buffer containing 1% mouse serum. After washing and further blocking, slides were incubated with secondary antibody for one hour at RT. DAPI (4′,6-diamidino-2-phenylindole) was used to visualize cell nuclei. The secondary antibody used in this section is anti-rat Alexa Fluor 594-conjugated antibody (Molecular Probes/Invitrogen). Primary antibodies are indicated in Suppl. Table 2.

For the detection of apoptotic cells, a TUNEL (terminal deoxynucleotidyl transferase dUTP nick end labeling) assay was performed by using the in situ cell death detection kit (Fluorescein, 11684795910, Roche). Analysis of quantification of positive cells was performed by using the open source software ImageJ.

### 2.8. Statistical Analysis

All results are expressed as mean ± SE and represent data from 5 animals per group. All significant *p* values were measured by one-way ANOVA test, followed by Bonferrroni's posttest for the comparison between groups. A value of *p* < 0.05 was considered significant (^∗^*p* < 0.05, ^∗∗^*p* < 0.01).

## 3. Results

### 3.1. Nrf2 Overexpression in Hepatocytes Lacking c-met Receptor Results in Reduced Triglycerides Accumulation upon MCD Feeding

**Figure 1:**
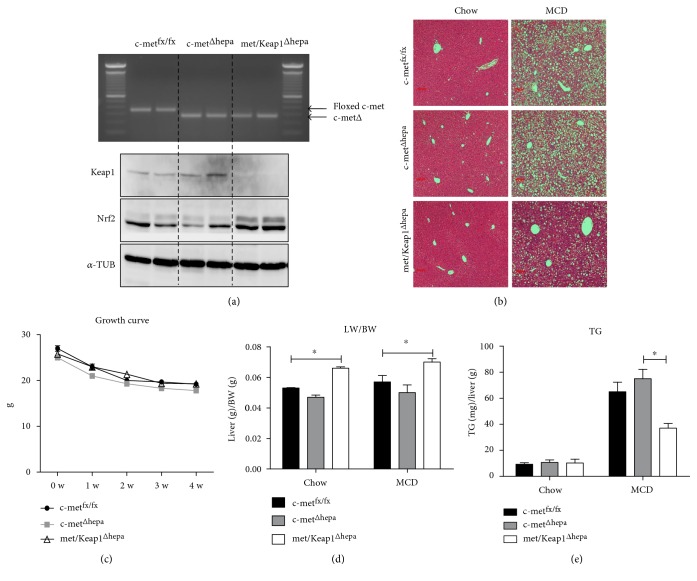
Genotyping of c-met gene performed on cDNA transcribed from hepatic RNA showing amplification of the floxed region of the gene with and without cre-recombinase activation ((a), upper panel). Western blot panel for Keap1 and Nrf2 protein expression analysis ((a), lower panels). Representative pictures of hematoxylin and eosin (H&E) staining on liver section of c-met^fx/fx^, c-met^Δhepa^, and met/Keap1^Δhepa^ mice after 4 weeks chow (left panels) and MCD (right panels) diet (b). Weekly growth curve of c-met^fx/fx^, c-met^Δhepa^, and met/Keap1^Δhepa^ mice during a 4-week course of MCD treatment. ^∗^Data are expressed as mean ± SE, Student's *t*-test with *p* < 0.05 (*N* = 5) (c). Liver body weight ratio (d) and intrahepatic triglyceride content measurement (e) after 4-week chow and MCD diet feeding. ^∗^Data are expressed as mean ± SE, ANOVA test with *p* < 0.05 (*N* = 5).

**Figure 2:**
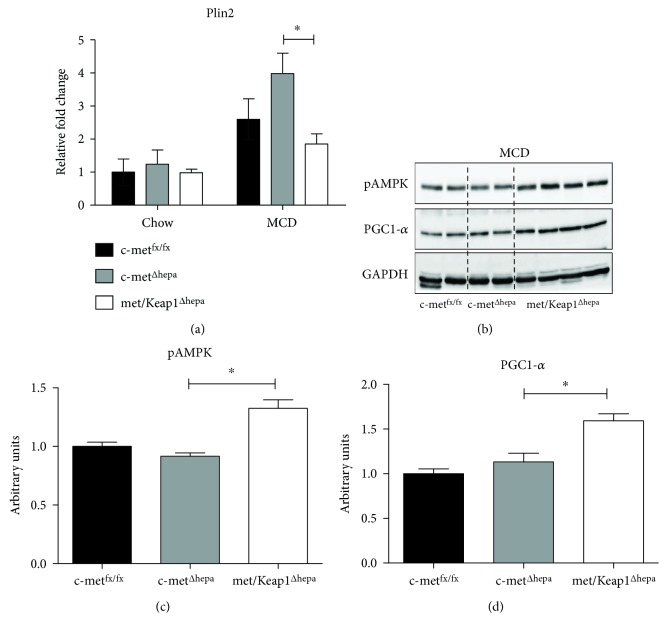
Analysis of hepatic gene expression of proteins involved in lipid droplet formation (a). Hepatic protein expression levels from Western blot (b) and densitometric analysis of band intensities (c and d) performed on total liver lysates of mice after 4 w MCD administration. ^∗^Data are expressed as mean ± SE, ANOVA test with *p* < 0.05 (*N* = 5).

As previously described, deletion of the exon 15 in c-met^Δhepa^ mice operated by the cre-recombinase under control of the albumin promoter resulted in a defective intracellular activation of the receptor as indicated by impaired phosphorylation of specific tyrosine residues (data not shown). The effective deletion of c-met exon 15 was confirmed by reverse transcriptase-PCR analysis (Figure 1(a), upper graph). As expected, selective hepatic deletion of Keap1 resulted in increased protein levels of the transcription factor Nrf2 (Figure 1(a), lower graph). This stabilization correlated with augmented transcriptional activity as assessed by expression of a well-known target gene Nqo1 (Suppl. Fig. 1a). Displaying no differences of total body weight, hepatocyte c-met deletion resulted in a slight reduction of the hepatic mass. Further, deletion of Keap1 induced a moderate but significant increase of liver weight as compared to control mice, under normal chow feeding, only subtly depending on changes of the cell proliferation rate (Suppl. Fig. 1b). However, microscopically, we could not detect any obvious alteration as emerged from histological analysis of hematoxylin and eosin (H&E) staining (Figure 1(b), left panels). After 4 weeks of MCD feeding, met/Keap1^Δhepa^ still displayed a significant increase of the liver mass without significant alterations of body weight that progressively declined in all groups (Figures 1(c) and 1(d)). Interestingly, double knockouts showed decreased accumulation of lipid droplets and a lower grade of steatosis as confirmed by histological analyses of H&E staining (Figure 1(b), right panels). Biochemical analyses revealed that met/Keap1^Δhepa^ accumulated about 50% less TG in the liver as compared to other experimental groups (Figure 1(e)). Accordingly, gene expression of the lipid droplet associated protein, Plin2, showed an absolute increase of steatohepatitis progression in c-met^Δhepa^ mice. In contrast, the same gene was significantly downregulated in met/Keap1^Δhepa^ hepatocytes as compared with the single mutants (Figure 2(a)). Furthermore, double knockout mice displayed increased hepatic expression and phosphorylation of AMPK and augmented expression of the transcription factor PGC-1*α* compared to the other experimental groups (Figures 2(b), 2(c), and 2(d)), indicating enhanced fatty acid oxidation and mitochondrial biogenesis. These results confirmed that Nrf2 overactivation is sufficient to enhance hepatic lipid catabolism and mitochondrial functionality as we recently illustrated in detail elsewhere [9].

### 3.2. Nrf2 Overexpression Dampens the Exacerbation of Oxidative Stress Production in Hepatocytes Lacking c-met Receptor upon MCD Feeding

**Figure 3:**
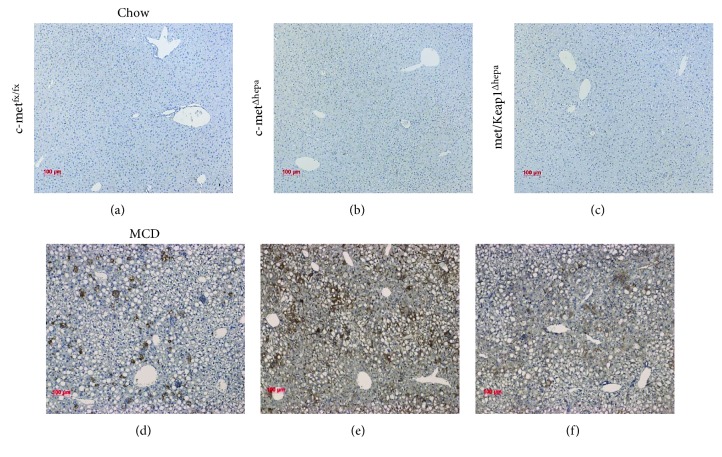
Immunohistochemistry staining for oxidative stress marker 4-HNE on liver sections of c-met^fx/fx^, c-met^Δhepa^, and met/Keap1^Δhepa^ mice after 4-week chow (a, b, c) and MCD (d, e, f) diet administration, respectively.

**Figure 4:**
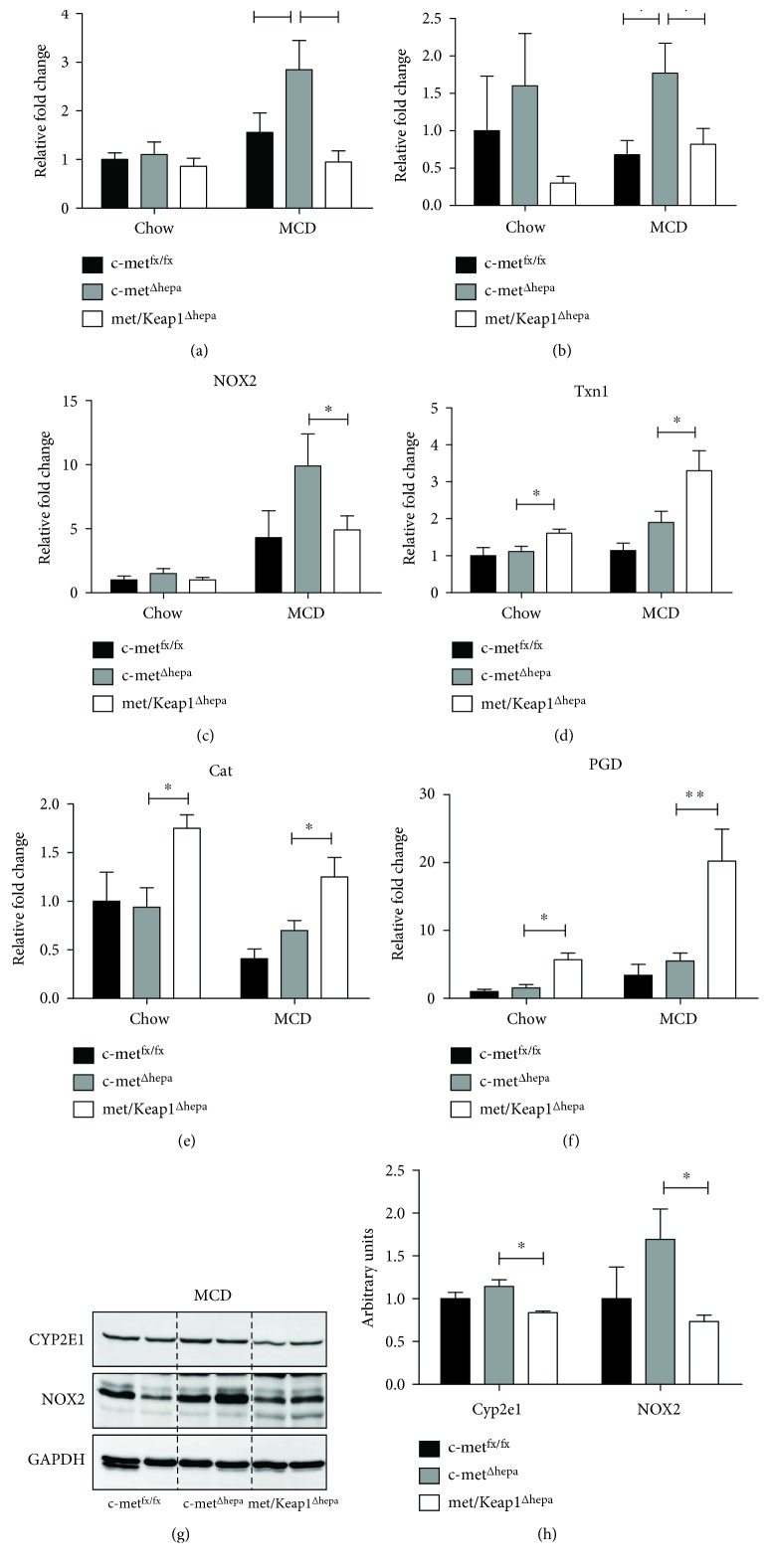
Analysis of hepatic gene expression of proteins involved ROS production (a, b, c) and of proteins responsible for the antioxidant cell defense (d, e). (f) ^∗∗^*p* values < 0.01. ^∗^Data are expressed as mean ± SE, ANOVA test with *p* < 0.05 (*N* = 5). Hepatic protein expression levels of Cyp2e1 and NOX2 from Western blot performed on total liver lysates of mice after 4 w MCD administration (g) with relative densitometric analysis (h).

Whereas under chow diet, oxidative stress was barely detectable, and after 4 weeks of MCD administration, hepatocyte-specific c-met deletion resulted in increased production of oxidative stress compared to wild type, as evidenced by immunostaining analysis of 4-HNE, a bioproduct of lipid peroxidation (Figures 3(a), 3(b), 3(c), 3(d), and 3(e)). In contrast, overactivation of Nrf2 induced by Keap1 deletion resulted in a strong decrease of 4-HNE-positive cells at levels even lower than the control group (c-met^fx/fx^) (Figure 3(f)). Of note, histological analyses indicated that met/Keap1^Δhepa^ livers not only showed a decreased number of 4-HNE positive cells but the intensity of the signal was also lower compared to c-met^fx/fx^ and single c-met^Δhepa^ (Figures 3(d), 3(e), and 3(f)). More importantly, as also observed in our previous microarray analyses [5], c-met deletion resulted in derepression of pro-oxidant enzymes directly responsible for the generation of cellular ROS, such as Cyp2e1, Cyp4a10, and the NADPH oxidase NOX2 (Figures 4(a), 4(b), and 4(c)). Thus, hepatic induction of these genes as well as the protein expression (Figures 4(g) and 4(h)) was dramatically blunted by overexpression of Nrf2 in met/Keap1^Δhepa^ mice. These results seem to offer further evidence for a role of HGF/c-met signaling in the preservation of the cellular redox balance. Furthermore, Nrf2 activation resulted in upregulation of gene expression of enzymes involved in H_2_O_2_ and free radical scavenging, such as catalase, thioredoxin-1, and the pentose phosphate pathway intermediate enzyme 6-phosphogluconate dehydrogenase (PGD) (Figures 4(d), 4(e), and 4(f)).

### 3.3. Nrf2 Overexpression Drastically Reduces the Number of Apoptotic Cells in Hepatocytes Lacking c-met Receptor upon MCD Feeding

**Figure 5:**
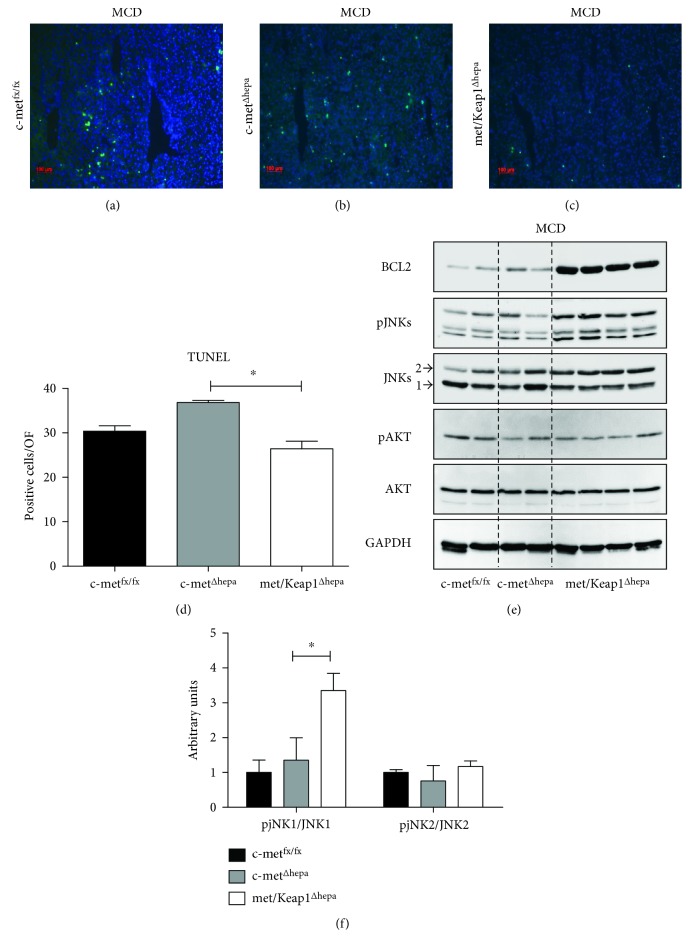
TUNEL immunofluorescence of c-met^fx/fx^ (a), c-met^Δhepa^ (b), and met/Keap1^Δhepa^ (c) liver sections (magnification 100x) after 4 weeks of MCD feeding, with relative morphometric analysis (d). ^∗^Data are expressed as mean ± SE, ANOVA test with *p* < 0.05 (*N* = 5). Hepatic protein expression levels from Western blot (e) and densitometric analysis of band intensities (f) performed on total liver lysates of mice after 4 w MCD administration.

In association with increased ROS production, TUNEL assay revealed that the number of apoptotic hepatocytes in c-met^Δhepa^ livers was augmented as compared to the other experimental groups (Figures 5(a), 5(b), 5(c), and 5(d)), as already observed in other experimental situations. Concomitant Keap1 deletion in c-met^Δhepa^ hepatocytes significantly turned down programmed cell death to levels comparable with the control group (Figure 5(d)) although no significant differences in the serum transaminase levels were detected (data not shown). As previously observed, Nrf2 activation correlated with a dramatic increase of the antiapoptotic protein Bcl-2 (Figure 5(e)). Surprisingly, phosphorylation levels of Akt were reduced in c-met^Δhepa^ as well as met/Keap1^Δhepa^ hepatocytes, ruling out an involvement of this pathway in Nrf2-mediated cell survival (Figure 5(e)). Interestingly, met/Keap1^Δhepa^ livers showed a significant increase of phosphorylation of the JNKs—specifically JNK1—kinases (Figures 5(e) and 5(f)) with no evident differences for other MAPK kinases such as ERK1/2 (data not shown).

### 3.4. Hepatic Nrf2 Overexpression in Livers Lacking c-met Receptor Decreases the Influx of Infiltrating Inflammatory Cells upon MCD Feeding

**Figure 6:**
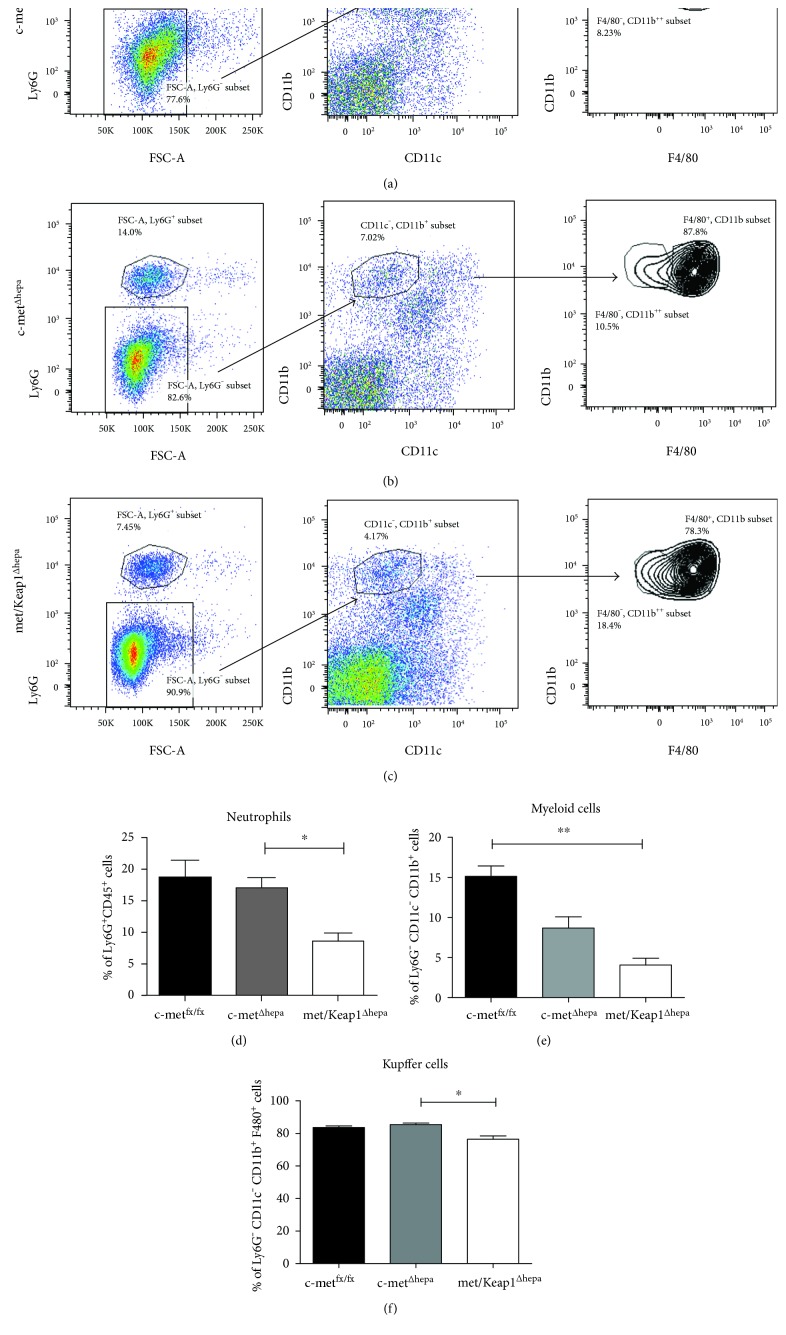
Representative gating strategy from flow cytometry analysis of intrahepatic neutrophils and monocyte/macrophage populations performed on total liver lysates of c-met^fx/fx^ (a), c-met^Δhepa^ (b), and met/Keap1^Δhepa^ (c) after 4 weeks of MCD feeding. Neutrophils were gated by FSC/SSC, CD45^+^/alive CD11b^+^, and Ly6G^+^ cells. Macrophages were gated by FSC/SSC, CD45^+^/alive, Ly6G^−^, CD11b^+^, F4/80^+^. Quantification of the gated populations reported on histogram (d, f). (e) ^∗∗^*p* values < 0.01. ^∗^Data are expressed as mean ± SE, ANOVA test with *p* < 0.05 (*N* = 3 − 5).

**Figure 7:**
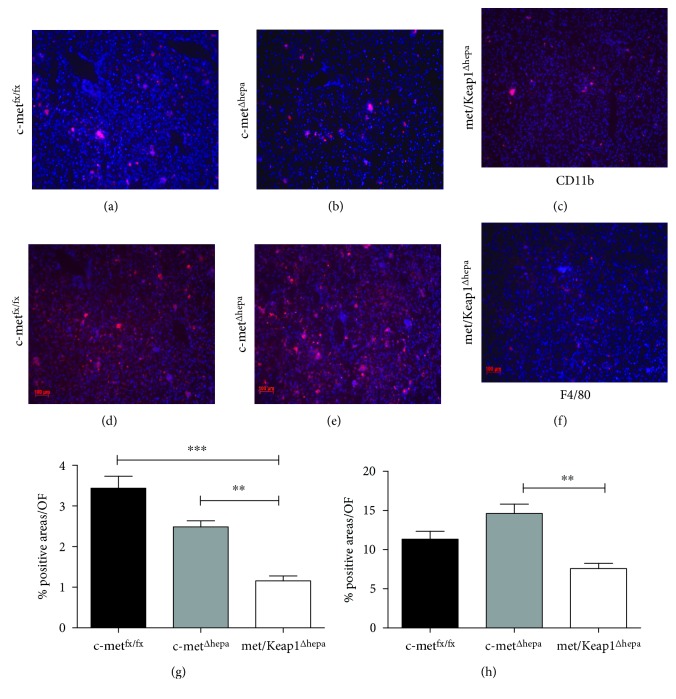
Immunofluorescence staining for neutrophil/monocyte marker CD11b (a, b, c) and macrophage marker F4/80 (d, e, f) of c-met^fx/fx^, c-met^Δhepa^, and met/Keap1^Δhepa^ liver sections (magnification 100x) after 4 weeks of MCD feeding with relative morphometric analyses (g) ^∗∗∗^*p* values < 0.01. (h) ^∗∗^*p* values < 0.01. ^∗^Data are expressed as mean ± SE, ANOVA test with *p* < 0.05 (*N* = 3 − 5).

FACS analysis performed on liver lysates after 4 weeks of MCD treatment revealed that met/Keap1^Δhepa^ accumulate less proinflammatory neutrophils compared to the other experimental groups, as indicated by the number of Ly6G^+^ cells (Figures 6(a), 6(b), 6(c), and 6(d)). Interestingly, the number of circulating neutrophils was also significantly reduced in these animals (Suppl. Fig. 3b). Similarly, the number of activated macrophages, measured as F4/80^+^/CD11b^+^ cells, was dramatically reduced in the livers of met/Keap1^Δhepa^ compared to the other groups. In line with these findings, hepatic gene expression of proinflammatory cytokines, Ccl-2 and TNF-*α*, was dramatically downregulated in met/Keap1^Δhepa^ mice (Suppl. Fig. 1c and data not shown). These results were confirmed by immunofluorescence analyses of CD11b-positive cells (Figures 7(a), 7(b), and 7(c)) and F4/80-positive macrophages (Figures 7(d), 7(e), and 7(f)) supported by morphometric quantifications (Figures 7(g) and 7(h)). Importantly, these histological pictures pointed out the dramatic reduction of the number of inflammatory cell clusters in met/Keap1^Δhepa^ livers after MCD administration. Surprisingly, met/Keap1^Δhepa^ mice showed a significant increase of CD4^+^ lymphocytes infiltrating into the liver whereas no changes in the CD8^+^ lymphocytes number were observed (Suppl. Fig. 2 and Suppl. Fig. 3a).

### 3.5. Nrf2 Overexpression in Hepatocytes Attenuates the Enhanced Development of Fibrosis Resulting from Hepatocyte-Specific c-met Deletion upon MCD Feeding

**Figure 8:**
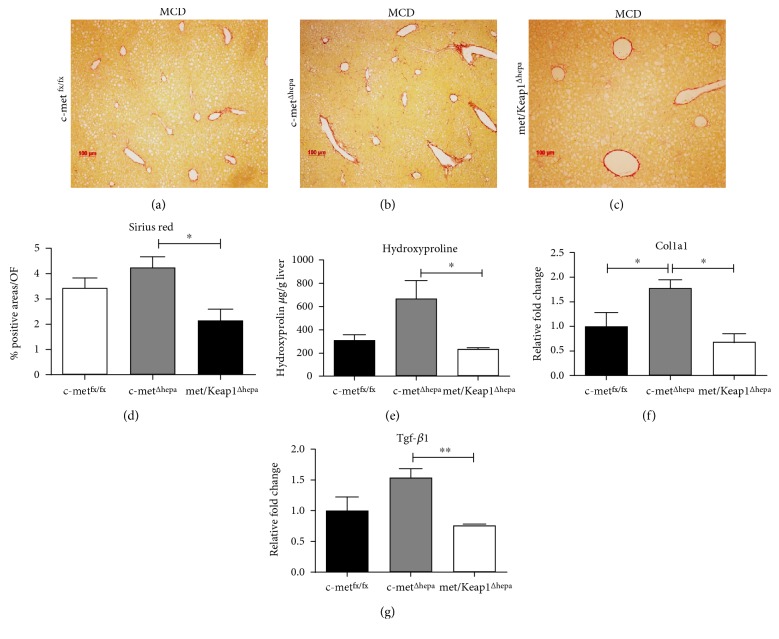
Sirius red staining of c-met^fx/fx^ (a), c-met^Δhepa^ (b), and met/Keap1^Δhepa^ (c) liver sections (magnification 100x) after 4 weeks of MCD feeding with relative morphometric analysis (d). Intrahepatic hydroxyproline content (e) and hepatic gene expression of profibrotic mediators (f) (g) ^∗∗^*p* values < 0.01, from livers of mice after 4 weeks of MCD feeding. ^∗^Data are expressed as mean ± SE, ANOVA test with *p* < 0.05 (*N* = 5).

As also described in our previous report [5], c-met deletion in hepatocytes accelerates appearance and progression of liver fibrosis in several models of chronic liver injury as well as by feeding a MCD diet. Single c-met knockout livers displayed an increased deposition of collagen fibers as observed in the Sirius red staining (Figures 8(a), 8(b), and 8(c)) and confirmed by related morphometric analysis (Figure 8(d)). This observation was further confirmed by biochemical measurement of intrahepatic hydroxyproline content indicating the highest collagen accumulation in c-met^Δhepa^ livers (Figure 8(e)). Interestingly, fibrosis was strongly inhibited in the double mutants where Nrf2 was overactivated. Thus, Sirius red staining and hydroxyproline content were suppressed to the levels of the control group (Figures 8(a), 8(b), 8(c), 8(d), and 8(e)). Concordantly, deletion of Keap1 in hepatocytes lacking c-met also restored the hepatic expression of profibrotic mediators as Col1A1 and TGF-*β*1 to control levels (Figures 8(f) and 8(g)).

## 4. Discussion

The generation of reactive oxidative species associated with mitochondrial alterations and the activation of pro-oxidant enzymes still represents an unsolved issue in the context of nonalcoholic steatohepatitis in which it seems to play a pivotal role in the exacerbation of liver injury, inflammation, and repair. Although the mitochondria represent the major source of free radicals already under physiological conditions, other cell compartments such as microsomes and lysosomes participate in ROS production via oxidative reactions under stressful conditions. Increased activity of the cytochrome p450, particularly of the isoform Cyp2e1, has emerged as an important free radical generator during NASH [13]. Indeed, a direct association between Cyp2e1 and Cyp4a10 enzyme expression and the initiation of lipid peroxidation during the progression of NASH has been clearly identified [14]. Moreover, a critical role for NADPH oxidases (such as NOX2) in the development of features related to aggravation of NASH and, in general, of the metabolic syndrome has been convincingly demonstrated by Garcia-Ruiz et al. [15]. Following these considerations, many experimental and clinical studies based on scavenging or buffering of free radicals have begun to show promising results in a more relevant therapeutic context [16, 17]. It is worth to mention that in the context of NASH several therapeutic options targeting oxidative stress via Nrf2 activation already achieved preclinical and clinical phase trials [18]. In particular, beyond classical natural antioxidant such as sulforaphane or resveratrol, the synthetic electrophilic compound oltipraz (dithiolethione) revealed promising effects in the treatment of liver metabolic diseases and is currently being analyzed in a phase II clinical trial.

The role of HGF/c-met axis in liver pathophysiology has been extensively investigated with a particular light on aspects regarding liver regeneration, hepatocyte proliferation, and apoptosis [19]. Moreover, activation of this signaling pathway has been repeatedly reported to exert hepatoprotective effects against experimental conditions characterized by oxidative stress [20, 21]. In isolated hepatocytes, Clavijo-Cornejo et al. showed that HGF exerts a biphasic regulation of the NADPH oxidases with a short-time effect inducing the activation of the enzyme and a long-time effect after which the persistence of a functional HGF/c-met pathway results to be necessary for the suppression of the NADPH oxidases components in an Nrf2-dependent manner [22]. In line with these findings, a recent work from our group [5] demonstrated that c-met-deleted hepatocytes displayed enhanced oxidative stress and increased apoptotic cell death in association with overproduction of superoxide anion in vivo. This led to enhanced progression of hepatic inflammation and fibrosis. Recently, Dominguez-Perez et al. showed that administration of HGF reduces hepatocyte susceptibility to lipotoxicity through an increase of antioxidant defenses such as *ϒ*-GCS and GSH thereby attenuating ROS formation and damage [23]. The generation of double mutant c-met/Keap1^Δhepa^ mice further demonstrated that re-establishing a functional antioxidant activity completely reversed the accelerated pathological conditions observed in single c-met^Δhepa^ mice. In particular, the reduction of oxidative stress was accompanied by a decrease of the abovementioned pro-oxidant systems, Cyp2e1, Cyp4a10, and NOX2 expression. Conversely, expression of antioxidant cell weapons, such as catalase and thioredoxin-1, in addition to the well-known activation of the pentose phosphate pathway, was strongly upregulated. It is interesting to note that the amelioration of the redox balance occurred concomitantly with a reduced hepatic accumulation of triglycerides related to the inhibition of the LXR-dependent lipogenic program induced by Nrf2 as previously shown [9, 24]. These findings actually consolidate our former data set illustrating reduced TG accumulation and oxidative stress in hepatocytes carrying a genetic activation of Nrf2 in two experimental models of diet-induced steatohepatitis [9]. Moreover, this aspect coherently matches with the activation of the AMPK/PGC-1*α* pathway observed in met/Keap1^Δhepa^ hepatocytes. Although a causal relation with Nrf2 activation remains poorly elucidated, Nrf2-dependent negative regulation of the lipogenic gene SCD-1 could contribute to hepatic AMPK activation as shown by Dobrzyn et al. [25]. Lastly, it would be reasonable to connect AMPK activation with maintenance of the NADPH production originating from the pentose phosphate pathway as an antioxidant mechanism in response to metabolic stress, as elegantly proposed elsewhere [26].

In relation to this observation, TUNEL-positive hepatocytes in double knockouts were strongly diminished as compared to those in control and single c-met knockouts. Interestingly, independent of the degree of steatosis, the loss of c-met impairs hepatocyte capacity to counteract oxidative stress generated by fatty acid oxidation, thereby sensitizing cells to apoptosis. This suggests that reducing oxidative stress via Nrf2 activation might represent a key step in the protection from programmed cell death as we already observed in hepatocyte-specific Keap1 knockout mice [10]. Curiously, in the present work, cell protection was not associated with enhanced activation of the PI3K/AKT survival pathway. Thus, a significant increase of the stress-activated JNK phosphorylation was observed. Although the importance of this point results unclear, JNKs have been recently shown to promote nuclear Nrf2 activation [27]. Moreover, it might be relevant to underline that liver size was not significantly affected by administration of MCD diet in all the experimental groups and the double knockouts already display hepatomegaly under normal conditions. Analyses of immunofluorescence for the proliferation marker Ki67 indicated that hepatocyte proliferation seems to contribute only minimally to this phenotype. Thus, we reasonably believe that under normal chow conditions hepatomegaly might be the result of a synergistic effect of proliferation and cellular hypertrophy. Upon MCD feeding, a compensative proliferation in response to liver injury and hepatocyte cell death is commonly activated. Single hepatocyte-specific c-met deletion constantly results in diminished proliferation rate being partly responsible for the impaired tissue repair as previously reported. Genetic Nrf2 overactivation does not seem to have an impact on the proliferation rate but the extent of cell death is indeed dramatically reduced. Therefore, in conditions of metabolic stress, the increase in liver/body weight ratio observed in double knockouts results to be maintained through reduced cell death and increased cell functionality (hypertrophy).

In line with these findings, simultaneous deletion of c-met and Keap1 resulted in a dramatic decrease of infiltrating neutrophils (Ly6G^+^) and inflammatory monocytes (CD11b^+^/F4/80^+^). This indicates a modulation of the immune response possibly driven by reduced liver injury and through the negative modulation of specific inflammatory mediators. Although the reduction of neutrophils might be beneficial in the context of NASH in terms of a decreased MPO release [28], on the other hand, further studies are required in order to identify the phenotype of monocyte subpopulations since the cell polarization might differently influence the progression of the disease. In contrast, livers of c-met/Keap1^Δhepa^ mice display a significant increase of infiltrating CD3^+^ lymphocytes, characterized by a predominant CD4^+^ subtype. These findings seem to be consistent with a recent study showing that, in the context of NASH, CD4^+^ lymphocytes seem to be more susceptible to fatty acid-induced ROS cytotoxicity [29]. Consequently, a reduction of CD4^+^ population results to be deleterious in the progression of fibrosis. This aspect is going to emerge as a reproducible variable in several experimental conditions associated with hepatic Nrf2 overexpression. Further investigations are currently in progress in order to shed light on the nature and functions of this hepatic infiltrate.

Finally, loss of c-met in hepatocytes was previously shown to accelerate the onset of fibrosis not only in several experimental murine models [30] but also in NASH [5], through multiple mechanisms involving increased hepatocyte cell death, altered release of inflammatory mediators, oxidative stress, and cell proliferation. However, production of ROS remains a central player in the pathogenesis of liver fibrosis even if the causal relation in the context of cell death and repair is still an argument of intense investigation [31]. Curiously, whereas overexpression of Nrf2 in c-met/Keap1^Δhepa^ resulted in less TG accumulation, less oxidative stress, and reduced number of inflammatory cells as compared to controls and single knockouts, the degree of fibrosis in these mice was only moderately dampened to the degree of the control group. These data indicate that genetic enhancement of Nrf2 signaling is sufficient to repress MCD-dependent oxidative stress and cell damage but the efficacy against fibrosis seems to be restricted to a limited spectrum of transcriptional activation. To this end, identification of immune mediators directly regulated through Nrf2 transcriptional activity could shed light on the dark side of chronic overexpression of this transcription factor.

## 5. Conclusion

The results reported in this work offer further evidence for Nrf2-mediated cytoprotection (partly illustrated also in conference data set [32]). They pinpoint a key role for HGF/c-met signaling in the regulation of redox homeostasis. Actually, genetic Nrf2 overexpression revealed therapeutic effects in c-met-deficient hepatocytes by counteracting oxidative stress thereby attenuating the disease progression. This work highlights critical aspects to be considered for the development of novel therapeutic strategies in the management of NASH.

## Supplementary Material

The information of supplementary materials are as follows: Supplementary Figure 1. q-PCR analysis of hepatic gene expression of the Nrf2 target gene Nqo1 (A) and morphometric analysis of Ki67+ hepatocytes from immunofluorescence staining (B). q-PCR analysis of hepatic gene expression of the the pro-inflammatory mediator Ccl2 (C). ∗Data are expressed as mean ± SE, ANOVA-Test with p<0.05 (N=5). Supplementary Figure 2. Representative gating strategy from flow cytometry analysis of lymphoid populations performed on total liver lysates of c-met^fx/fx^ (A), c-met^Δhepa^ (B) and met/keap1^Δhepa^ (C) after 4 weeks of MCD feeding. Total lymphocytes were gated by FSC/SSC, CD45+/alive CD3^+^, NK1.1^-^ cells and respective CD3^+^, CD4^+^ subpopulation. Supplementary Figure 3. Quantification of the hepatic lymphoid populations (A) and of the blood myeloid population (B) reported on histogram. ∗Data are expressed as mean ± SE, ANOVA-Test with p<0.05 (N=3–5). Supplementary Figure 4. Schematic overview of the proposed mechanisms. The hepatic increase of fatty acid accumulation in form of triglycerides (TG) results in boosting the intracellular oxidative stress production that through oxidative modification of Keap1 might contribute to Nrf2 stabilization. Interestingly, oxidative stress has also been proposed to inhibit HGF/c-met signaling. In turn, c-met activation certainly triggers cyto-protective pathways that directly or indirectly regulate the cellular oxidative balance. Transcriptomic data indicate a probable cross talk between Keap1/Nrf2 axis and HGF/c-met pathway, although the specific relation is still poorly understood. Suppl. Table 1. qPCR primers used in this study. Suppl. Table 2. Antibodies used in this study.
