# Supplementary figures and images for: Genetic Nrf2 Overactivation Inhibits the Deleterious Effects Induced by Hepatocyte-Specific c-met Deletion during the Progression of NASH
Source: Oxid Med Cell Longev. 2017 Jun 6;2017:3420286. doi: 10.1155/2017/3420286 (PMC5476895; doi:10.1155/2017/3420286)

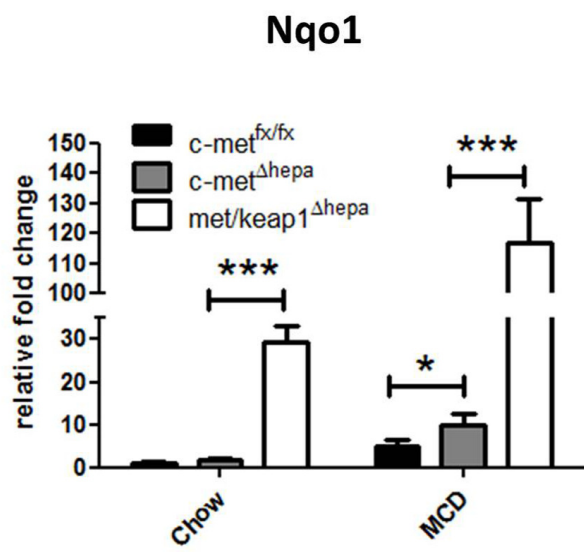

A.

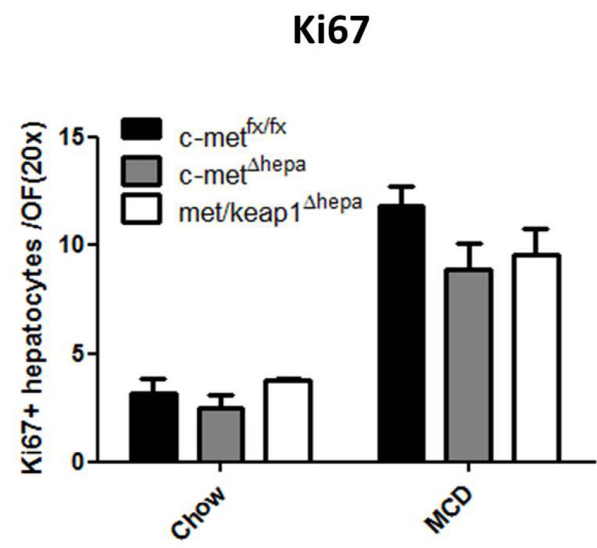

B.

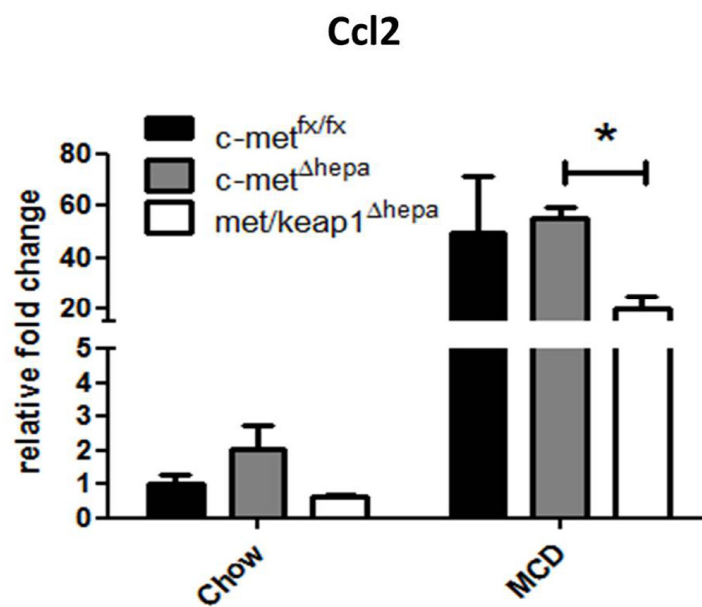

C.

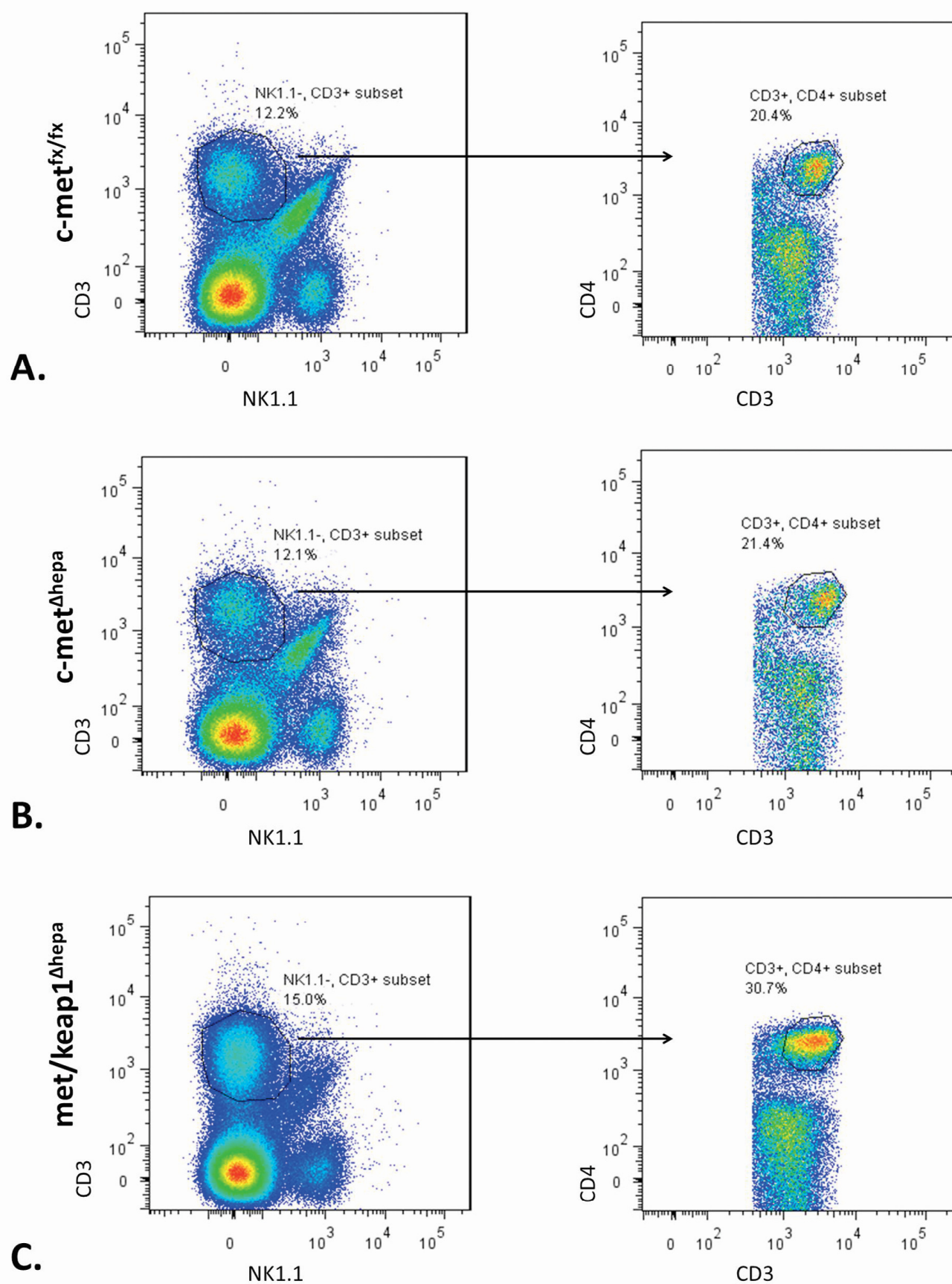

## Liver FACS

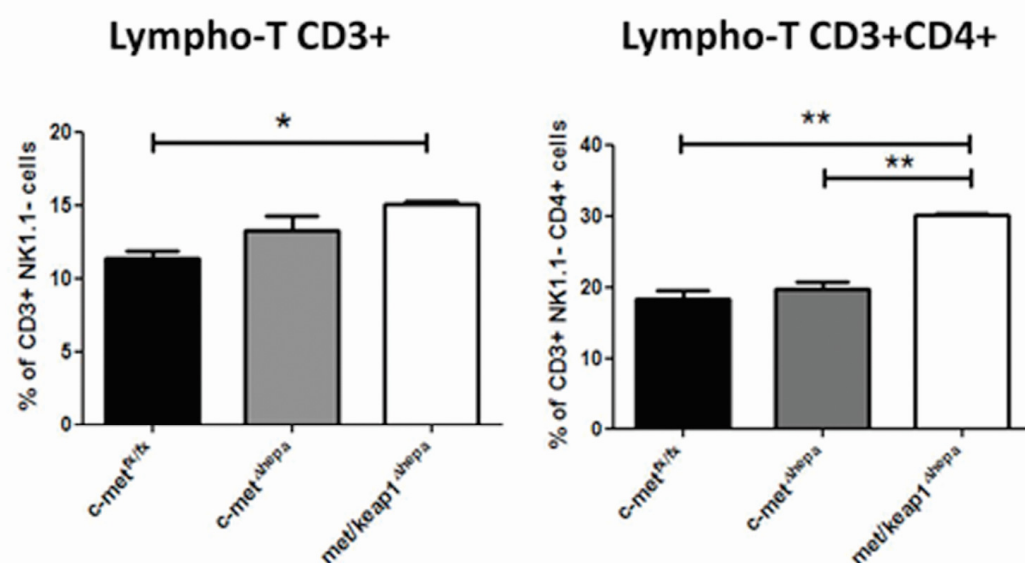

A.

## Blood FACS

## Neutrophils

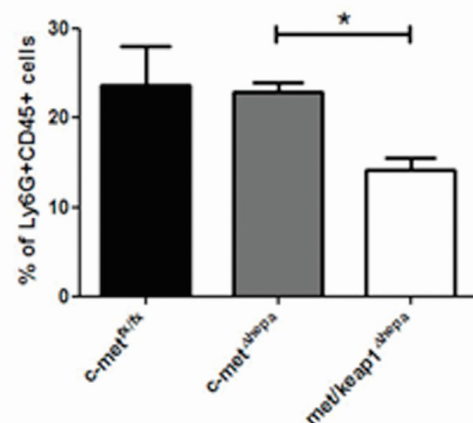

## Macrophages

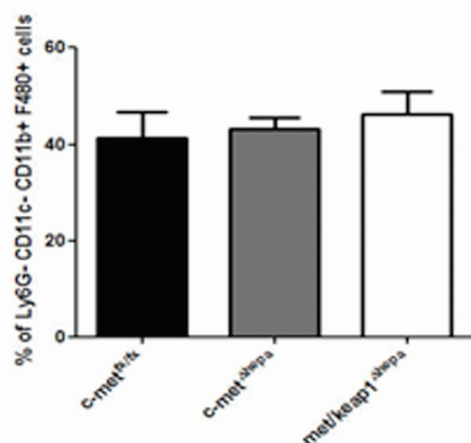

## Monocytes

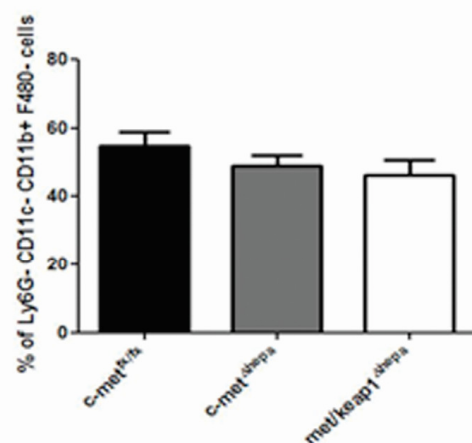

B.

Suppl. Figure 4

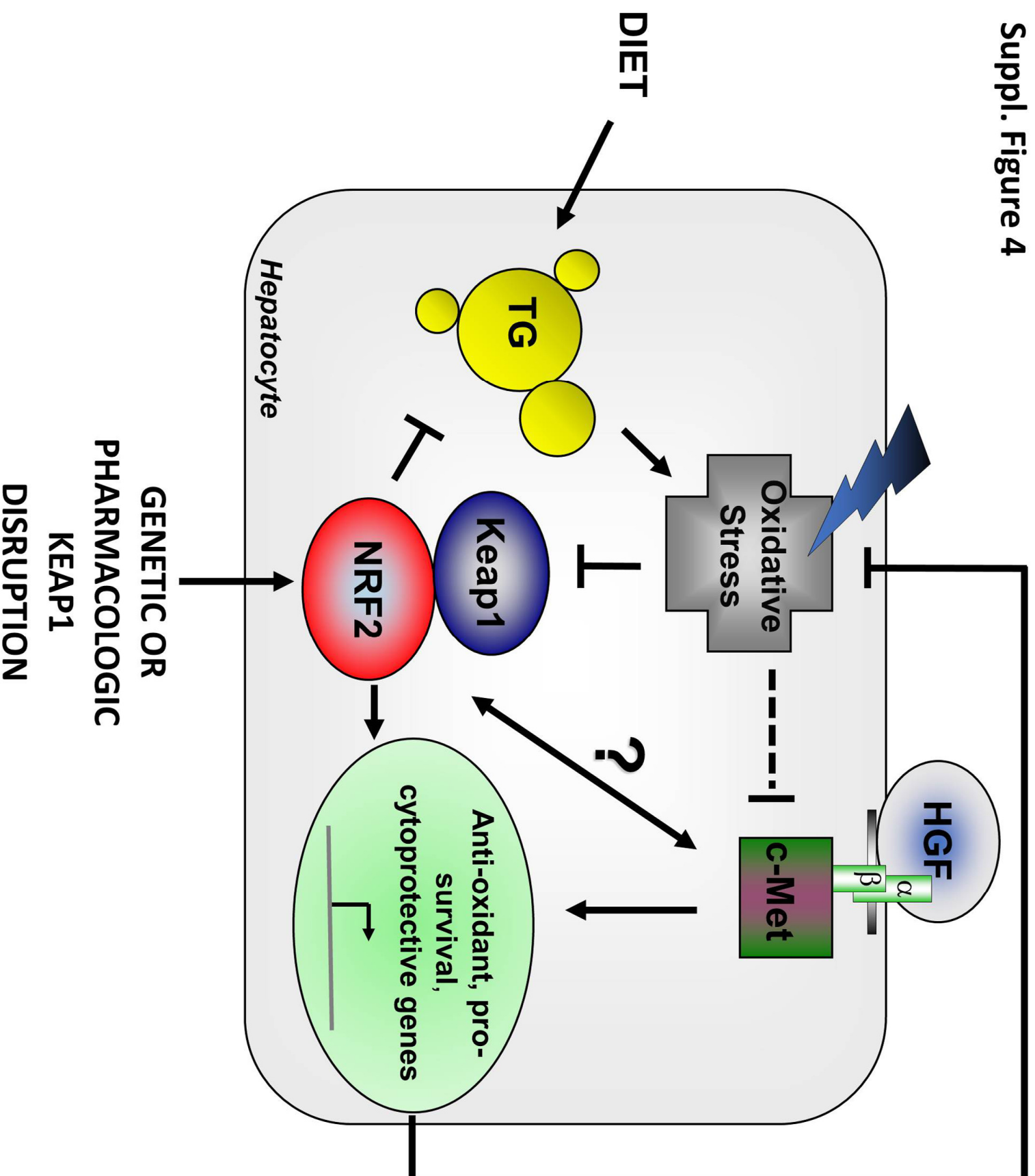

Supplement: Supplementary file 1 — The information of supplementary materials are as follows: Supplementary Figure 1. q-PCR analysis of hepatic gene expression of the Nrf2 target gene Nqo1 (A) and morphometric analysis of Ki67+ hepatocytes from immunofluorescence staining (B). q-PCR analysis of hepatic gene expression of the the pro-inflammatory mediator Ccl2 (C). ∗Data are expressed as mean ± SE, ANOVA-Test with p<0.05 (N=5). Supplementary Figure 2. Representative gating strategy from flow cytometry analysis of lymphoid populations performed on total liver lysates of c-metfx/fx (A), c-metΔhepa (B) and met/keap1Δhepa (C) after 4 weeks of MCD feeding. Total lymphocytes were gated by FSC/SSC, CD45+/alive CD3+, NK1.1- cells and respective CD3+, CD4+ subpopulation. Supplementary Figure 3. Quantification of the hepatic lymphoid populations (A) and of the blood myeloid population (B) reported on histogram. ∗Data are expressed as mean ± SE, ANOVA-Test with p<0.05 (N=3–5). Supplementary Figure 4. Schematic overview of the proposed mechanisms. The hepatic increase of fatty acid accumulation in form of triglycerides (TG) results in boosting the intracellular oxidative stress production that through oxidative modification of Keap1 might contribute to Nrf2 stabilization. Interestingly, oxidative stress has also been proposed to inhibit HGF/c-met signaling. In turn, c-met activation certainly triggers cyto-protective pathways that directly or indirectly regulate the cellular oxidative balance. Transcriptomic data indicate a probable cross talk between Keap1/Nrf2 axis and HGF/c-met pathway, although the specific relation is still poorly understood. Suppl. Table 1. qPCR primers used in this study. Suppl. Table 2. Antibodies used in this study. [file 3420286.f1.pdf]
