# Supplementary material for: Genetic Nrf2 Overactivation Inhibits the Deleterious Effects Induced by Hepatocyte-Specific c-met Deletion during the Progression of NASH
Source: Oxid Med Cell Longev. 2017 Jun 6;2017:3420286. doi: 10.1155/2017/3420286 (PMC5476895; doi:10.1155/2017/3420286)
Supplement: Supplementary file 2 [file 3420286.f2.docx]

**Suppl. Table I. qPCR primers used in this study**

|  | **Fwd Sequence** | **Rev Sequence** |
| --- | --- | --- |
| **Mmu Plin2** | GTCCACCTGATTGAATTCGC | CGATGTGCTCAACACAGTG |
| **Mmu Cat** | TGAGAAGCCTAAGAACGCAATTC | CCTTCGCAGCCATGTGAGA |
| **Mmu Nox2** | GAGCTGAACGAATTGTACGTG | TCCATTTCCAAGTCATAGGAGG |
| **Mmu Scd-1** | GTTCCAGAATGACGTGTACGA | GGCTTGTAGTACCTCCTCTG |
| **Mmu Cyp2e1** | TTTAACCAAGTTGGCAAAGCG | CCCTTGTTCTTGTACTCCTGG |
| **Mmu Cyp4a10** | CCAAATCCAGAGGTGTTTGAC | ATTGTTTCCCAATGCAGTTCC |
| **Mmu Nqo1** | TGACATCACAGGTGAGCTG | ACCACTGCAATGGGAACTG |
| **Mmu Txn1** | ACTGCCAGGATGTTGCTG | TTCCTTGTTAGCACCGGAG |
| **Mmu Pgd** | AACAAAGAGGCTTGGCCC | TCTTCAAATGCCTGAGCCA |
| **Mmu Tgfβ1** | TTGCTTCAGCTCCACAGAG | AAGGACCTTGCTGTACTGTG |
| **Mmu Mcp-1** | TCAGCCAGATGCAGTTAACG | AACTACAGCTTCTTTGGGACAC |
| **Mmu Col-1a1** | GCTACTACCGGGCCGATGATGC | CCTTCGGGGCTGCGGATGTTC |
| **Mmu 18S** | GTAACCCGTTGAACCCCATT | CCATCCAATCGGTAGTAGCG |

**Suppl. Table II. Antibodies used in this study.**

| **Antibody** | **HOST** | **Application** | **Dilution** | **Company** |
| --- | --- | --- | --- | --- |
| **F4/80** | rat | IF | 1/100 | AbD Serotec |
| **CD11b** | rat | IF | 1/100 | BD Pharmingen |
| **4-HNE** | mouse | IH | 1/200 | Abcam |
| **AKT** | rabbit | WB | 1/1000 | Cell Signaling |
| **pAKT (Ser473)** | rabbit | WB | 1/1000 | Cell Signaling |
| **pAMPK (Thr172)** | rabbit | WB | 1/1000 | Cell Signaling |
| **Nrf2** | rabbit | WB | 1/1000 | Gene Tex |
| **pERK1/2 (Thr202/Tyr204)** | rabbit | WB | 1/1000 | Cell Signaling |
| **pJNK (Thr183/Tyr185)** | rabbit | WB | 1/1000 | Cell Signaling |
| **JNKs** | rabbit | WB | 1/1000 | Cell Signaling |
| **GAPDH** | mouse | WB | 1/10000 | AbD Serotec |
| **α-Tubulin** | mouse | WB | 1/1000 | Sigma Aldrich |
| **NOX2** | rabbit | WB | 1/1000 | Gene Tex |
